# Muscle formation during embryogenesis of the polychaete *Ophryotrocha diadema *(Dorvilleidae) – new insights into annelid muscle patterns

**DOI:** 10.1186/1742-9994-5-1

**Published:** 2008-01-02

**Authors:** Annette Bergter, John L Brubacher, Achim Paululat

**Affiliations:** 1 Department of Zoology, University of Osnabrueck, Barbarastr. 11, D-49069 Osnabrueck, Germany; 2 Department of Biological Sciences, University of Manitoba, Winnipeg, MB, R3T 2N2, Canada

## Abstract

**Background:**

The standard textbook information that annelid musculature consists of oligochaete-like outer circular and inner longitudinal muscle-layers has recently been called into question by observations of a variety of complex muscle systems in numerous polychaete taxa. To clarify the ancestral muscle arrangement in this taxon, we compared myogenetic patterns during embryogenesis of *Ophryotrocha diadema *with available data on oligochaete and polychaete myogenesis. This work addresses the conflicting views on the ground pattern of annelids, and adds to our knowledge of the evolution of lophotrochozoan taxa.

**Results:**

Somatic musculature in *Ophryotrocha diadema *can be classified into the trunk, prostomial/peristomial, and parapodial muscle complexes. The trunk muscles comprise strong bilateral pairs of distinct dorsal and ventral longitudinal strands. The latter are the first to differentiate during myogenesis. They originate within the peristomium and grow posteriorly through the continuous addition of myocytes. Later, the longitudinal muscles also expand anteriorly and form a complex arrangement of prostomial muscles. Four embryonic parapodia differentiate in an anterior-to-posterior progression, significantly contributing to the somatic musculature. Several diagonal and transverse muscles are present dorsally. Some of the latter are situated external to the longitudinal muscles, which implies they are homologous to the circular muscles of oligochaetes. These circular fibers are only weakly developed, and do not appear to form complete muscle circles.

**Conclusion:**

Comparison of embryonic muscle patterns showed distinct similarities between myogenetic processes in *Ophryotrocha diadema *and those of oligochaete species, which allows us to relate the diverse adult muscle arrangements of these annelid taxa to each other. These findings provide significant clues for the interpretation of evolutionary changes in annelid musculature.

## Background

Recent molecular and morphological studies have dramatically changed our understanding of the phylogenetic relationships between major taxonomic divisions of bilaterally symmetrical animals (Bilateria). Most bilaterian taxa are now grouped into three major clades: deuterostomes, ecdysozoans and lophotrochozoans [1,2]. This phylogenetic rearrangement has stimulated new scientific interest in lophotrochozoan taxa in particular, as this group of phyla has lacked well-studied model organisms. In order to address general evolutionary questions about Bilateria, we need to better understand the morphology and embryonic development of lophotrochozoan taxa, such as annelids [3-12].

Our work focuses on the comparative analysis of annelid muscle formation during embryogenesis, in order to expand the developmental and morphological knowledge base for annelids, and consequently, for Lophotrochozoa in general [6-8]. We studied myogenesis using cLSM (confocal laser scanning microscopy) analysis of fluorescently labeled phalloidin, which binds to F-actin of muscles. This approach has proved to be a powerful method to study muscular arrangements in many soft-bodied taxa [13-18].

The ground pattern of the annelid musculature is generally assumed to comprise an outer layer of homogeneous circular and an inner layer of longitudinal muscles, as is characteristic for oligochaete annelids. This model was originally developed in the 19th century, and has become well-established textbook information [19-23]. A corollary of this model is the theory that the ancestral annelid stem species was a soil-dwelling animal in which such muscles, along with other morphological characteristics, would have facilitated burrowing into the soil by peristaltic movement [24-27]. This view of the basal annelid has, however, been recently challenged by proposals that the ancestral annelid was an aquatic, epibenthic animal, bearing parapodia – that is, polychaete-like [28,29].

Recent cLSM analyses, particularly of polychaete muscle patterns, have attempted to resolve these contradictory understandings of the evolutionary history of annelids. This work has clearly revealed that the homogenous circular and longitudinal muscle layers characteristic of oligochaetes are rarely found in polychaetes. Instead, a variety of other muscles exist in polychaetes in addition to longitudinal and circular fibers, including parapodial, diagonal, dorsoventral, chaetal, bracing and oblique muscles (the latter passing through the body cavity) [30-33]. Longitudinal muscles in polychaetes are arranged in distinct bands separated by considerable lateral gaps. Ventrally, they comprise two prominent ventrolateral muscle bands, plus a thin medial band just dorsal to the ventral nerve cord [33]. The number of dorsal longitudinal muscles is more variable, but one bilateral pair seems to be the most common pattern [34]. Complete circular muscles can be found in species of Glyceridae, Capitellidae, Maldanidae, and Arenicolidae [33,34], but circular muscles bands are incomplete in Nereididae and even absent in species of Aphroditidae, Chrysopetalidae, Nerillidae, Pisionidae, Spionidae and Opheliidae [32,33]. In *Dorvillea kastjani *(Dorvilleidae) the circular muscles are restricted to the spaces between the parapodia [31].

The complex muscle patterns of polychaetes and the uniform muscle layers present in oligochaetes apparently bear little resemblance to each other, which hampers direct comparison of these muscle systems. However, our studies on myogenesis in the oligochaetes *Enchytraeus coronatus *(Enchytraeidae) and *Limnodrilus *sp. (Tubificidae), revealed some similarities between muscle patterns in oligochaete embryos and those of adult polychaetes [6,7]. Both oligochaete species displayed distinct dorsal and ventral bilateral pairs of muscle strands at the onset of muscle formation, similar to the longitudinal muscles characteristic of polychaetes. The characteristic complete layer of longitudinal muscles in adults of these two species forms later during development, by addition of secondary strands. Initially, circular muscles form ventrolaterally in a repetitive pattern at the posterior margin of posterior body segments.

It is therefore apparent that comparative studies of muscle development in annelids (rather than adult musculature alone) may identify characteristic features of oligochaetes and polychaetes that could significantly improve our understanding of muscle arrangement in annelids, and annelid evolution [7,34]. Current annelid phylogeny remains poorly resolved, which considerably hampers our ability to draw conclusions about potential plesiomorphic states in annelids [2,21,28,29,35-39]. A solid knowledge of morphology and development from a diverse sampling of annelid taxa will deepen our knowledge of plesiomorphic or apomorphic characteristics. This new information, in combination with a reliable annelid phylogeny, will enable us to interpret and determine the direction of evolutionary events in this important group.

### Ophryotrocha diadema

**Figure 1:**
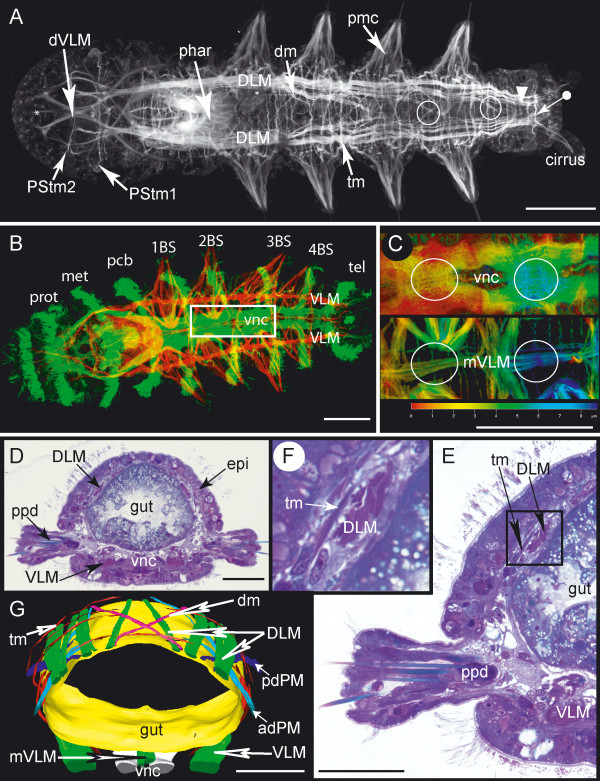
**Overview of musculature of *Ophryotrocha diadema***. Scale bars = 50 μm, anterior is to the left. **A**. Dorsal view, phalloidin staining. The most prominent muscles in the prostomium are the ventral diagonal muscles (dVLM). Two transverse muscles are present in the prostomium: PStm2 crosses the dVLM dorsally, whereas PStm1 lies between the dorsal and the ventral longitudinal muscles. The complex musculature of the ventrally situated pharynx (phar) is heavily stained. The inner strands of the dorsal longitudinal muscles (DLM) cross repeatedly at the dorsal midline (circles). Four pairs of parapodia are present, with prominent parapodial muscle complexes (pmc). Transverse (tm) and diagonal muscles (dm) cross the dorsal side irregularly. Several transverse muscles surround the posterior zone of segment proliferation (arrowhead). The last transverse muscle circle marks the pygidium (tagged arrow). **B**. Ventral view, phalloidin staining (red) and anti-acetylated-tubulin staining (green). The ciliary bands are stained by anti-acetylated tubulin antibody. These are the prototroch (prot) of the prostomium, the metatroch (met) and the ciliary band of the segment like annulus within the peristomium (pcb), and the four ciliary bands of the chaetigers (1–4 BS) anterior to the telotroch (tel) of the pygidium. The boxed region marks the close-up depicted in Figure C (vnc-ventral nerve cord, VLM-ventral longitudinal muscle) **C**. Close-up from B. Ventral view, phalloidin staining and anti-acetylated tubulin antibody depth coded (in μm). Upper panel shows the ventral nerve cord (vnc), lower panel shows the median ventral longitudinal muscle (mVLM). Depth coding indicates that the mVLM is situated dorsal (deep) to the vnc (circles). **D**. Histological cross section through chaetiger of an adult. The dorsal longitudinal muscles (DLM) lie just below the epidermis (epi). The uniramous parapodia (ppd) are ventrolaterally situated. The ventral longitudinal muscles (VLM) lie on either side of the ventral nerve cord (vnc). **E**. Close up from D. Same labeling as in D, additionally labeled are the dorsally situated transverse muscles (tm). **F**. Close up from E depicting the dorsal longitudinal muscle (DLM) and a transverse muscle (tm) lying above it (supralongitudinally). **G**. 3D-reconstruction from a series of histological sections in the same body-region as shown in D. Anterior is toward the top of the figure. Depicted are the dorsal longitudinal muscles (DLM) with their dorsomedian crossing strands (arrow), diagonal muscles (dm) and transverse muscles (tm) in a supralongitudinal position. The ventral longitudinal muscles (VLM) and the median ventral longitudinal muscle (mVLM) surround the ventral nerve cord (vnc – only the main tracts of the neuropile shown here). The anterior and posterior dorsal parapodial muscles (adPM, pdPM) stretch diagonally toward posterior and anterior respectively, underneath the dorsal longitudinal muscles.

The primary aim of this work is to compare patterns of myogenesis in a polychaete species with those known to occur in oligochaetes, in order to help clarify the ancestral muscle pattern of annelids. The morphology and life history of *Ophryotrocha diadema *(Dorvilleidae), previously described by Bertil Åkesson [40], resembles the postulated polychaete-like stem species described above. These are small, interstitial worms that inhabit shallow marine environments. Adults of this species grow to a maximum length of 4–5 mm, generally with fewer than 25 segments. The head is flattened and bears one dorsal pair of small antennae. Two ciliary bands, the acrotroch and prototroch, mark the prostomium. Posterior to the stomodeum are two additional rings of cilia belonging to the peristomium, the anterior one the metatroch. The second ring encircles a segment-like annulus of the peristomium, which lacks parapodia (unlike a "true" segment), and is characteristic of *Ophryotrocha *[41,42]. Each chaetae-bearing body segment, (chaetiger), is surrounded by a ciliary band, which persist throughout life and are used for swimming. The parapodia are uniramous and simple, without any lobes or cirri. They possess aciculae (specialized internal chaetae) and a small number of chaetae (Fig. 1D,F). The pygidium bears a pair of cirri and the dorsally situated anus.

Embryos of *O. diadema *are lecithotrophic, and develop within an egg case, lacking a free-swimming larval stage. Although embryos of *O. diadema *pass through a stage similar to the trochophore larva characteristic of polychaetes, this is not a free-living stage, nor do a number of chaetigerous segments arise simultaneously through metamorphosis, as in polychaetes with free-living trochophores [3,43]. Instead, development within the egg case proceeds with the formation of four chaetigerous segments one by one, prior to hatching. Thus, *O. diadema *undergoes direct development, and lacks a need for special larval musculature. This trait facilitates comparison with patterns of muscle development in the investigated oligochaetes, which likewise develop directly within a cocoon, and lack a free-living larval stage.

## Results

### Adult musculature

Embryos were classified into developmental stages according to the number of ciliary bands and chaetigers rather than time post-fertilization, as the timing of developmental events can vary considerably with environmental conditions and parental brooding (own observation). To provide information about the 3-dimensional arrangement of the musculature, we used depth-coded false-color cLSM projections.

#### Trunk musculature

**Figure 2:**
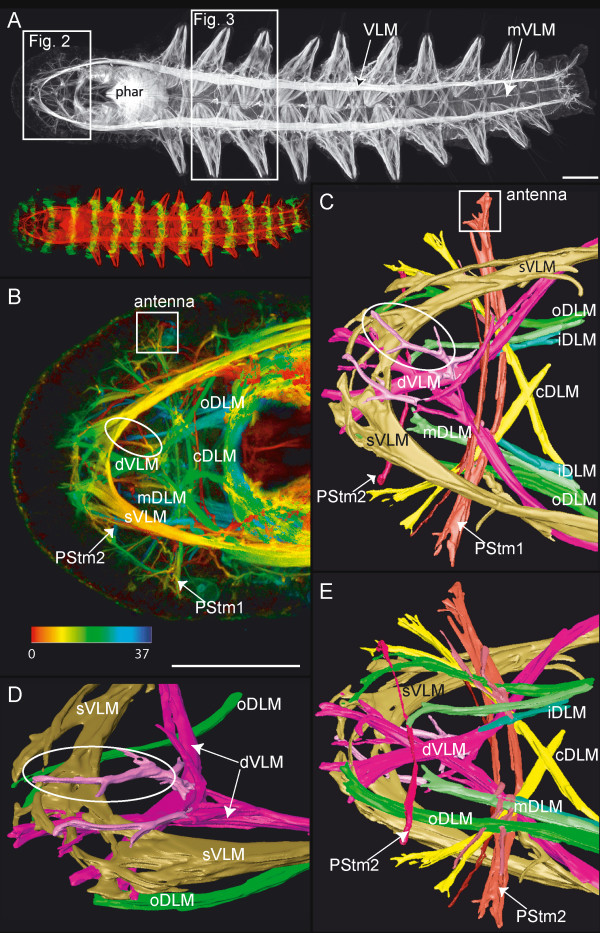
**Prostomial muscles**. Scale bars = 50 μm, anterior is to the left. **A**. Adult specimen with ten chaetigers: ventral view, phalloidin staining. Inset shows phalloidin staining (red) and anti-acetylated tubulin staining (green). Frames mark the regions used for 3D-reconstruction in panels B-E and Figure 3. Pharynx (phar), ventral longitudinal muscle (VLM) and median ventral longitudinal muscle (mVLM). **B**. Close up of frame depicted in A, phalloidin staining, depth coded (in μm). The prominent straight ventral longitudinal muscles (sVLM) and the diagonal ventral longitudinal muscles (dVLM) connect anteriorly with the outer dorsal longitudinal muscles (oDLM). A small muscle strand splits from each dVLM muscle and extends ventroanteriorly (circle). The most central pair of longitudinal muscle strands cross each other and run toward the opposite lateral side of the prostomium (cDLM). A transverse muscle (PStm1) lies between the dorsal and ventral longitudinal muscles. One part of the PStm1 reaches into the antennae (square). PStm2 – prostomial transverse muscle 2. **C – E**. 3D-reconstruction of muscles from the area depicted in Figure B. Labels are as follows: sVLM – straight ventral longitudinal muscle, dVLM – diagonal ventral longitudinal muscle, with ventroanteriorly running muscle portion (circle), PStm1 – prostomial transverse muscle 1, contributing to the antennae (square), PStm2 – prostomial transverse muscle 2, lying dorsal to the longitudinal muscles, oDLM, mDLM, iDLM – outer, middle, and inner straight-running branches of dorsal longitudinal muscle, cDLM – central pair of dorsal longitudinal branches, crossing each other. **C**. Ventral view. **D**. Ventroanterior view. **E**. Dorsal view.

The prominent musculature of the trunk in *Ophryotrocha diadema *comprises two bilateral sets of longitudinal muscles, the dorsal longitudinal muscles (DLM) and the ventral longitudinal muscles (VLM) (Fig 1A,B,DF and Fig. 2A). The dorsal longitudinal muscle is composed of distinguishable muscle strands. Of these, the innermost strand runs diagonally towards the dorsal midline where it crosses its bilateral counterpart (Fig. 1A, circle). This pattern is repeated posterior from the second chaetiger onwards. The longest longitudinal muscle strands reach as far as a transverse muscle anterior of the dorsally situated anus (Fig. 1A, tagged arrow).

**Figure 3:**
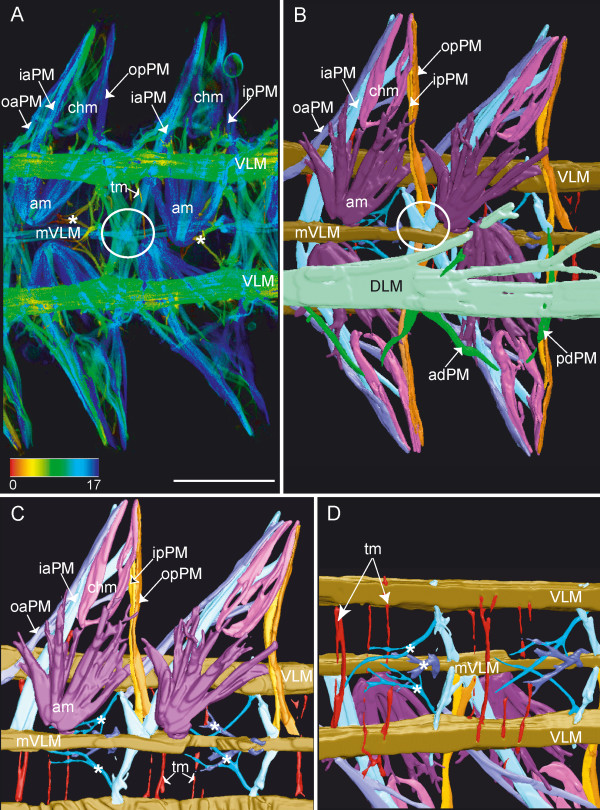
**Parapodial muscles**. Scale bars = 50 μm, anterior is to the left. **A**. Ventral view, close up from Fig. 2A, phalloidin staining, depth coded (in μm). Shown are two parapodia and their muscle complexes (pmc). The ventral longitudinal muscles (VLM) lie laterally, and the median ventral longitudinal muscle (mVLM) runs along the midline. Parapodial muscles are only labeled on the right half of the body. These include chaetal muscles (chm); acicular muscles (am); inner anterior parapodial muscle (iaPM); outer anterior parapodial muscle (oaPM); inner posterior parapodial muscle (ipPM); outer posterior parapodial muscle (opPM); median ventral longitudinal muscle (mVLM); the ipPM, opPM, iaPM assemble with the mVLM (circle). Thin transverse muscle strands (tm) cross the ventral side. **B**. 3D-reconstruction of A, but from dorsal side, same labeling scheme. Each muscle is displayed in a unique color. The dorsal longitudinal muscle (DLM) and the anterior (adPM) and posterior dorsal parapodial muscles (pdPM) have been removed from the reconstruction of the right side of the body, to expose underlying muscles. Chaetal (chm) and acicular muscles (am) form the center of the parapodium. The outer anterior parapodial muscle (oaPM) stretches anteriorly, terminating in the vicinity of the VLM. The inner anterior parapodial muscle crosses the body-cavity and ends at the opposite side (iaPM). Both posterior parapodial muscles (ipPM, opPM) terminate at the midline in close proximity to the median ventral longitudinal muscle (mVLM). Here, the ipPM, opPM, iaPM of two adjoining pairs of parapodial muscle complexes assemble (circle). **C**. 3D-reconstruction of A, dorsal view, with still more overlying muscles removed to expose ventral musculature. The consecutive parapodial muscle complexes of the right body-half are shown. Labeling as in B. Three muscles, originating from either of the ventral longitudinal muscles (VLM, mVLM) stretch ventroanteriorly (asterisks). Single transverse muscle strands (tm) of the ventral side are visible. **D**. Same 3D-reconstruction as in C. Ventral view.

The ventral longitudinal muscles (VLM) are formed by a thick assemblage of muscle fibers ventrolaterally, separated by a medial gap (Fig. 1D,F–G and Fig. 2A). Each muscle strand runs straight from the anterior pole to the posterior pole, decreasing in thickness, and terminating within the cirri or the pygidium. An additional, considerably thinner longitudinal muscle (mVLM) runs down the midline, just dorsal to the ventral nerve cord (vnc) (Fig. 1B,C,G and Fig. 2A). Transverse (tm) and diagonal muscles (dm) are evenly distributed over the dorsal side, running either below or above the longitudinal muscles (Fig. 1A,E–G). Some of these dorsal diagonal muscles are part of the parapodial muscle complex (Fig. 1G, adPM, pdPM, described below); others span the body laterally from the dorsal towards the ventral longitudinal muscle either anterior or posterior to the parapodia. On the ventral side, diagonal muscles are absent and transverse muscles are only weakly developed (Fig. 3D, tm). The only transverse fibers found here are present in the anterior portion of a body-segment. Three small muscles stretch from each of the ventral longitudinal muscles (VLM, mVLM) toward the anterior ventral region of each body-segment (Fig. 3A,C–D, asterisk).

#### Prostomial and peristomial musculature

The peristomium is dominated by strongly developed pharyngeal muscles (phar). They form a ventrally situated basket-like structure consisting of several layers in which the muscle fibers run in various directions, the outermost layer consisting of longitudinal fibers (Fig. 1A and 2A).

Most prostomial muscles arise from anterior elongations of the dorsal and ventral longitudinal muscles, forming a complex network in the head of the worm. All muscles described here are bilaterally paired unless otherwise noted, and are depicted in Figure 2B–E. The ventral portion of the ventral longitudinal muscles (VLM) extends straight toward the anterior pole of the prostomium; therefore we have termed these the straight ventral longitudinal muscles (sVLM). At the anterior, they separate into several flattened bands, each connecting with its bilateral partner. The dorsal portions of each ventral longitudinal muscle (VLM) separate at the anterior border of the peristomium and run diagonally (dVLM), crossing each other midway to the anterior pole. Here they connect with the straight ventral longitudinal muscles (sVLM). A pair of delicate longitudinal muscles originates from the diagonal ventral longitudinal muscles (dVLM) at their intersection point. These small muscles project ventrally towards the anterior pole, branching repeatedly along the way (Fig. 2B–D, circles).

Just anterior to the pharyngeal muscle complex, the dorsal longitudinal muscle (DLM) separates into four main strands. The three outer strands extend directly to the anterior. Of these three, the outer dorsal strand (oDLM) reaches the farthest and connects with the straight ventral longitudinal muscles (sVLM) and the diagonal ventral longitudinal muscles (dVLM), forming an anterior muscle cap (Fig. 2D). The middle dorsal longitudinal muscle (mDLM) is shorter and ends three-quarters of the way into the prostomium, while the inner muscle strand (iDLM) terminates about one-third of the way into the prostomium. The innermost (central) dorsal strand (cDLM) crosses toward the opposite side, intersecting with its bilateral counterpart.

One major prostomial transverse muscle (PStm1) crosses just anterior to the intersecting central dorsal longitudinal muscles (cDLM), running between the dorsal and ventral longitudinal muscles. One branch of this transverse muscle ends within the antennae. A second transverse muscle (PStm2) is thinner than the first prostomial transverse muscle (PStm1), and is situated dorsal to the dorsal longitudinal muscles.

#### Parapodial musculature

The majority of muscles present within the body segments belong to the parapodial muscle complexes. One such muscle complex is depicted in Figure 3. In the distal part of the parapodium, the chaetae are surrounded by chaetal muscles (chm), which form a long and slender crown of numerous thin muscle strands. A similar muscle crown encloses the aciculae. These special bristles do not penetrate the body wall, but support the chaetae. The acicular muscles (am) are strongly developed, originating from the base of the aciculae near the midline, and reaching into the proximal portion of the parapodium. There, the muscles divide into several filiform strands. The chaetal and the acicular muscles do not appear to have direct contact with each other.

The muscles responsible for the movement of the parapodia are the outer anterior and posterior parapodial muscles (oaPM and opPM, respectively) as well as the inner anterior and posterior parapodial muscles (iaPM and ipPM). These muscles terminate at the distalmost region of the parapodium. The outer anterior muscle (oaPM) is the shortest. It originates in the region of the ventral longitudinal muscle, whereas the inner anterior muscle (iaPM) crosses the ventral midline, having its origin at the opposite ventral longitudinal muscle. Both anterior muscles branch at the base of the parapodium. These secondary branches run diagonally – ventrally, distally and posteriorly from the chaetal muscles.

The posterior parapodial muscles both proceed straight to the ventral midline of the body, where they terminate. The posterior parapodial muscles intersect with the inner anterior muscle of the subsequent segment at a single point on the ventral midline, along with the median ventral longitudinal muscle (Fig. 3A,B, circle). Less prominent are a set of anterior and posterior dorsal parapodial muscles (adPM, pdPM) which enter the parapodium from the dorsal side (Fig. 3B and Fig. 1G). They do not reach the distal tip of the parapodium but end halfway down its length.

### Muscle formation during embryogenesis

#### Onset of muscle formation

**Figure 4:**
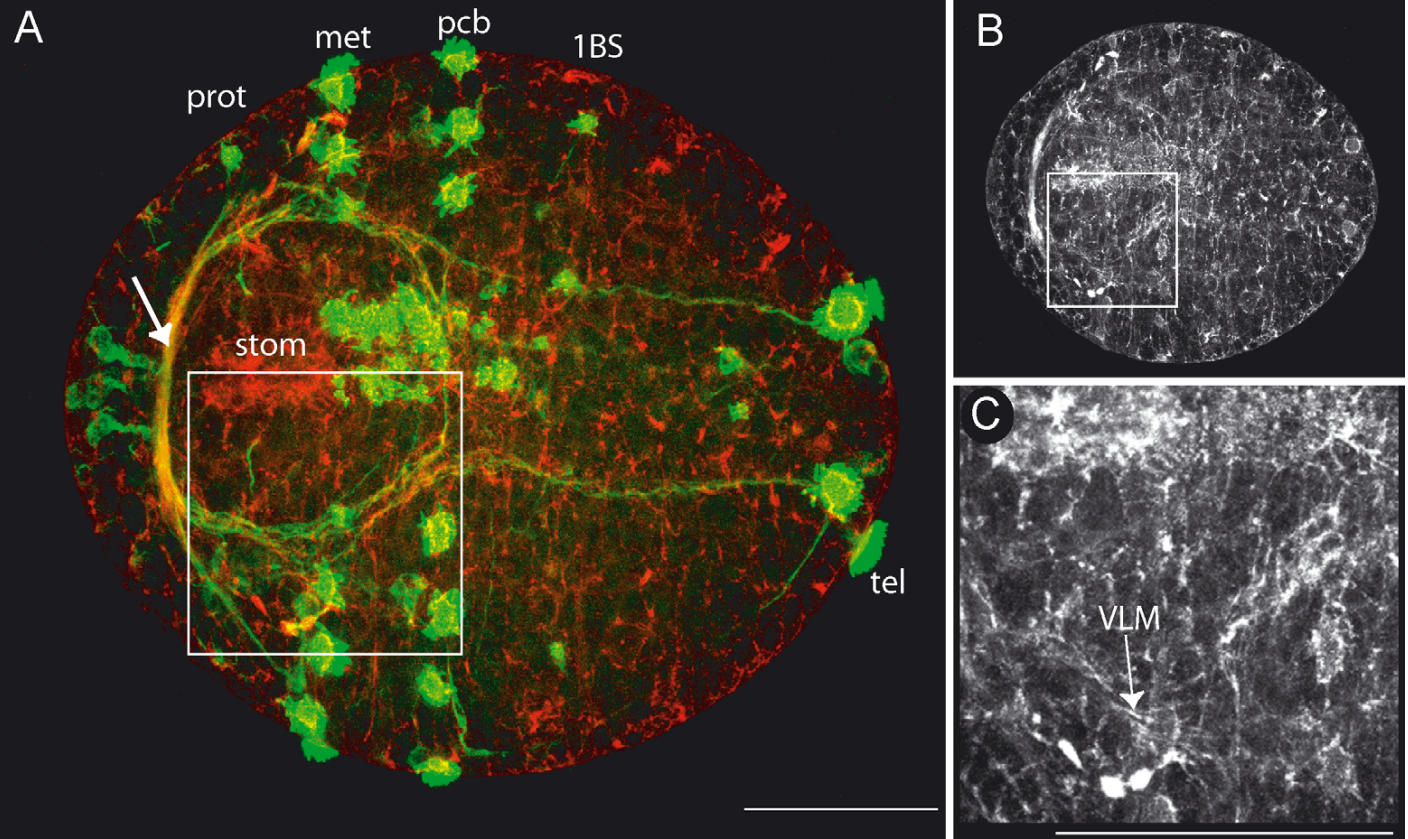
**Onset of muscle formation: early first chaetiger ciliary band**. Scale bars = 50 μm, anterior is to the left. **A**. Ventral view, phalloidin staining (red) and anti-acetylated tubulin staining (green). The anlage of the brain (arrow) and the circumoesophageal connectives surround the mouth opening (stom). Prototroch (prot), metatroch (met), the peristomial (pcb) and the first chaetiger ciliary band (1BS) and the telotroch (tel) are stained by the anti-acetylated tubulin antibody. The square marks the region of the close up depicted in C. **B**. Same picture as A, but only phalloidin staining. At this stage, phalloidin prominently labels the F-actin of the cytoskeleton, marking all cell margins. **C**. Close up from A, phalloidin staining only. The first discernable muscle fibers of the ventral longitudinal muscle (VLM) become apparent.

The first discernable muscle fibers become apparent after gastrulation has taken place, during a stage that appears very similar to a trochophore larva, despite the fact that *O. diadema *has no free-living larval stage. At this point, the major landmarks on the embryo are the ciliary bands and developing nervous system. From anterior to posterior, 4 ciliary bands are clearly visible: the prototroch (prot), metatroch (met), an additional peristomial ciliary band (pcb) and the telotroch (tel) of the pygidium. The first ciliary band (1BS) of the anteriormost body segment is present, but only weakly developed (Fig. 4A). Posterior to the metatroch, a longitudinally directed ciliary band – known as the neurotroch or gastrotroch – extends along the ventral midline of the embryo. The anlage of the brain (Fig. 4A, arrow), with its circumoesophageal connectives and the first commissure, form a circle around the mouth opening (stom) (Fig. 4A). Adjacent to the lateral margins of the mouth opening, the first fibers of the ventral longitudinal muscles (VLM) become visible (Fig. 4A–C).

#### One chaetigerous ciliary band

**Figure 5:**
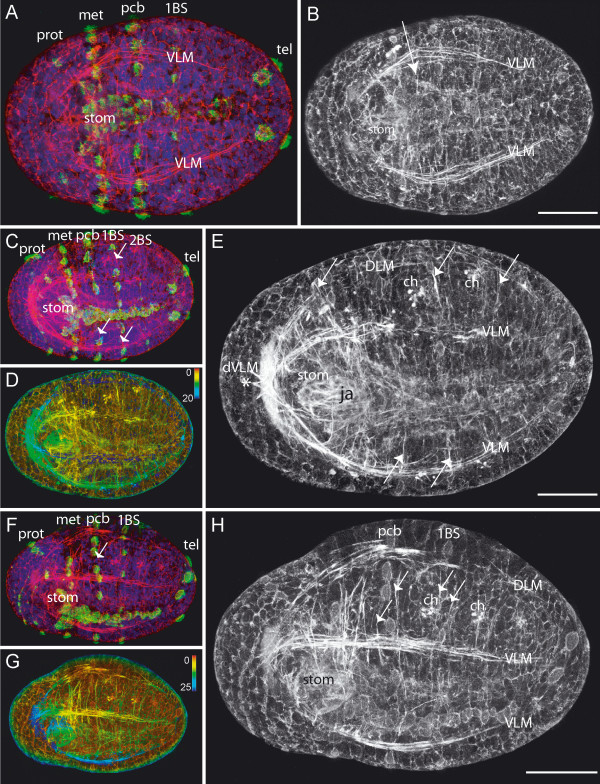
**First chaetiger ciliary band – later stage**. Scale bars = 50 μm, anterior is to the left. **A, C, F**. Phalloidin staining shown in red anti-acetylated tubulin staining in green, and nuclear staining in blue. Prototroch (prot), metatroch (met), ciliary band of peristomium (pcb), ciliary bands of chaetigers (BS) and telotroch (tel). Additional labeling as in **B, E, H**. Phalloidin staining. **D, G**. Phalloidin staining, depth coded (in μm). **A**. Same embryo as in **B**. Ventral view. The ventral longitudinal muscles (VLM) have elongated towards the pygidium. The first transverse muscles (arrow) differentiate. Mouth region (stom) clearly visible. **C, D**. Same Embryo as in **E**. Ventrolateral view. The dorsal (DLM) and ventral longitudinal muscles (VLM) have reached the pygidium. Adjacent to the autofluorescent chaetae (ch) lie transverse muscles, the anlage of the paraopodial muscles, laterally and ventrally (arrows). The ventral longitudinal muscles extend into the prostomium, forming three projections (dVLM, asterisk). The jaw apparatus (ja) is apparent ventral to the mouth opening (stom). **F, G**. Same Embryo as in **H**. Ventrolateral view. One transverse parapodial muscle anlage (arrow) is always present just anterior and just posterior of a ciliary band (pcb, BS). DLM-dorsal longitudinal muscle, VLM-ventral longitudinal muscle, ch-chaetae, stom-mouth opening.

With time, the embryo lengthens and the mandibles of the jaw apparatus (ja) become discernable due to weak autofluorescence (Fig 5E). The dorsal and ventral longitudinal muscle strands (DLM, VLM) elongate towards the posterior, reaching the pygidium at the end of this developmental stage (Fig. 5B,E,H). They consist of distinct fiber bundles, which decrease in thickness from anterior-to-posterior. The pair of ventral longitudinal muscles forms an anterior loop, with three terminal spikes at the ventral midline in front of the mouth opening (Fig. 5E). These represent the first muscles of the protostome, which correspond with the diagonally running ventral longitudinal muscles (dVLM) mentioned above (Fig. 2B–D).

**Figure 6:**
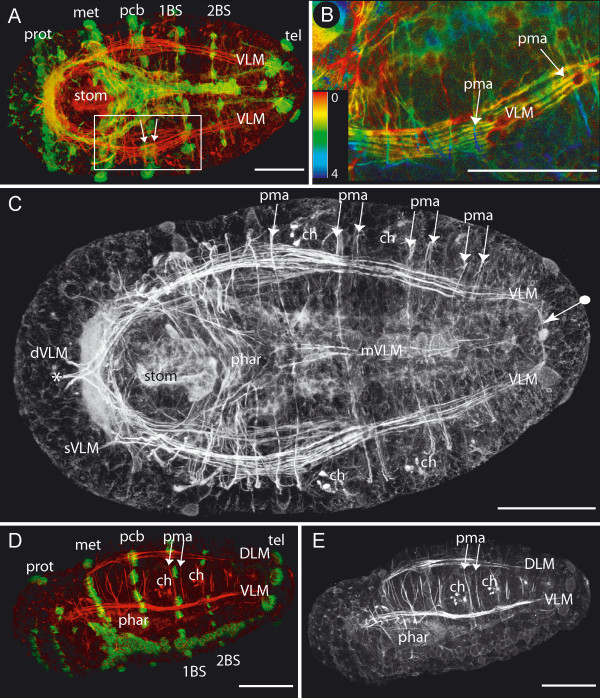
**Two chaetigerous ciliary bands**. Scale bars = 50 μm, anterior is to the left. **A-E**. Phalloidin staining (red) and in **A, D **anti-acetylated tubulin staining (green). Prototroch (prot), metatroch (met), ciliary band of peristomium (pcb), ciliary bands of chaetigers (BS) and telotroch (tel). **A**. Ventral view. Square marks region depicted in B. Labeling as in C. **B**. Close-up from A, ventral view, phalloidin staining, depth coded (in μm). The parapodial muscle anlage (pma) adjacent to the ciliary bands lie sublongitudinally, i.e., internal to the ventral longitudinal muscle (VLM). **C**. Same embryo as in A. Ventral view, phalloidin staining. The median ventral longitudinal muscle has now formed (mVLM). Its anterior splits, with branches running around each side of the pharyngeal muscles (phar). The ventral longitudinal muscles (VLM) project into the prostomium as the diagonal ventral longitudinal muscles (dVLM) and the straight ventral longitudinal muscles (sVLM) **D**. Lateral view. The parapodial muscle anlage (pma) runs on the lateral side of the embryo towards posterior. Each ciliary band (BS) lies between a set of parapodial muscle anlagen (pma). Autofluorescent chaetae (ch) are visible. **E**. Same embryo as in D. Lateral view, phalloidin staining only. The parapodial muscle anlagen (pma) adjacent to ciliary bands span from the ventral longitudinal muscle (VLM) dorsally to the dorsal longitudinal muscle (DLM). The musculature of the pharynx (phar) exhibits a basket-like shape.

At this stage, the first transverse muscles of the body segments begin to differentiate ventrally, in close proximity to the ventral longitudinal muscles (Fig. 5E,H, arrows). They extend towards the dorsal longitudinal muscles and the ventral midline, and are situated in a sublongitudinal position, i.e. deep to the longitudinal muscles (Fig. 6B) and are the anlage of the anterior and posterior parapodial muscles (pma). These muscles display a repetitive pattern with respect to the primordial body segments, such that a transverse fiber is always present directly anterior and another directly posterior to a ciliary band (Fig. 5B–H and Fig. 6A–E, arrows). Early signs of (autofluorescent) chaetae are already visible in what will become first three body segments. These emerge just anterior to chaetigerous ciliary bands, which mark the posterior margins of body segments at this developmental stage (Fig. 5H). Chaetae develop in an anterior to posterior progression, so the more posterior ones represent an earlier stage of development than those in anterior segments.

#### Two chaetigerous ciliary bands

As the body of the embryo continues to elongate, the ciliary band of the second body segment becomes recognizable (Fig. 6A). A fully circular muscle also forms within the pygidium at this time (Fig. 6C, tagged arrow), which marks the posteriormost extent of the dorsal and ventral longitudinal muscles.

The median ventral longitudinal muscle (mVLM) differentiates along the ventral midline. Unlike the lateral ventral longitudinal muscles (VLMs), the median ventral longitudinal muscle (mVLM) appears not to grow from anterior to posterior, but instead coalesces from primordia at various points along the AP axis (Fig. 6C). Anteriorly, its muscle fibers form a V that laterally encloses the mouth opening, and interlaces with the muscle fibers of the pharyngeal muscle complex (phar).

The initial thin fibers of the pharyngeal muscles already display the basketlike shape of the adult and range from the mouth opening to the anterior border of the first body-segment (Fig. 6E). The diagonal ventral longitudinal muscles of the prostomium (dVLM) have elongated farther to the anterior (Fig. 6C). Additionally, the straight-running ventral longitudinal muscles (sVLM) elongate from the ventral longitudinal muscles in the trunk towards the anterior pole, entering the prostomium on each side (Fig. 6C).

#### Three to four chaetigerous ciliary bands

**Figure 7:**
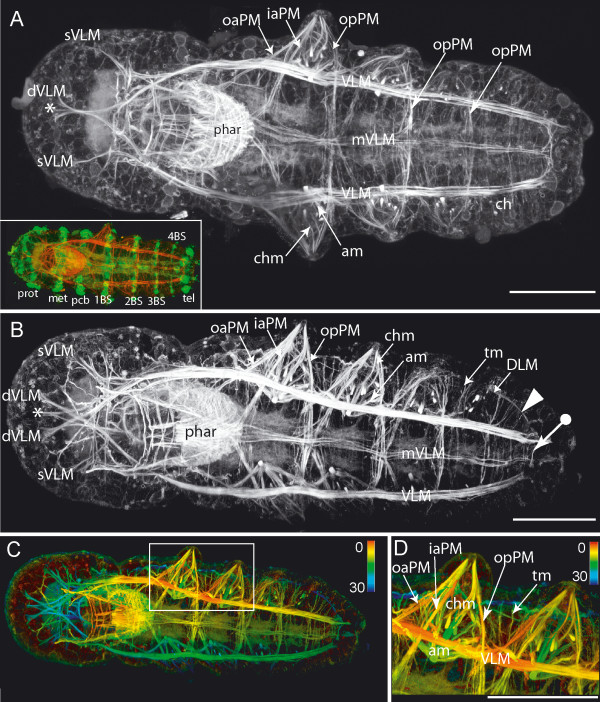
**Three to four chaetigerous ciliary bands: ventral view**. Scale bars = 50 μm, anterior is to the left. **A-D**. Phalloidin staining (inset of A shows phalloidin in red, and anti-acetylated tubulin staining in green) **A**. Ventral view. The ventral straight (sVLM) and diagonal prostomial muscles (dVLM) reach into the prostomium. The inner projection of the dVLM (asterisk) is less developed than the lateral projections. The pharyngeal muscles (phar) are heavily stained. The muscles of the first three parapodia (forming sequentially) are evident, and illustrate the developmental sequence. The most posterior parapodium shows only the outer posterior parapodial muscle (opPM) at this point, whereas the parapodia of the first chaetiger display the complete adult musculature: outer (oaPM) and inner anterior parapodial muscles (iaPM), chaetal muscles (chm), acicular muscles (am). The outer (opPM) and inner posterior parapodial muscle (ipPM) are not individually discernable. The chaetae of the fourth chaetiger (ch) are already visible. **B**. Ventrolateral view, phalloidin staining. Labeling as in A. Dorsal longitudinal muscle (DLM). Between this segment and the transverse muscle ring of the pygidium (tagged arrow) several transverse muscles are present within the zone of segment proliferation (open arrow). Ventral longitudinal muscle (VLM), median ventral longitudinal muscle (mVLM). **C**. Same as in B, phalloidin staining, depth coded (in μm). **D**. Close up from C, phalloidin staining, depth coded. Muscles of the parapodium: outer (oaPM) and inner anterior parapodial muscle), outer/inner posterior parapodial muscle (opPM), chaetal muscle (chm), acicular muscle (am) and ventral longitudinal muscle (VLM). Chaetal (chm) and acicular muscles (am) are still in close proximity.

**Figure 8:**
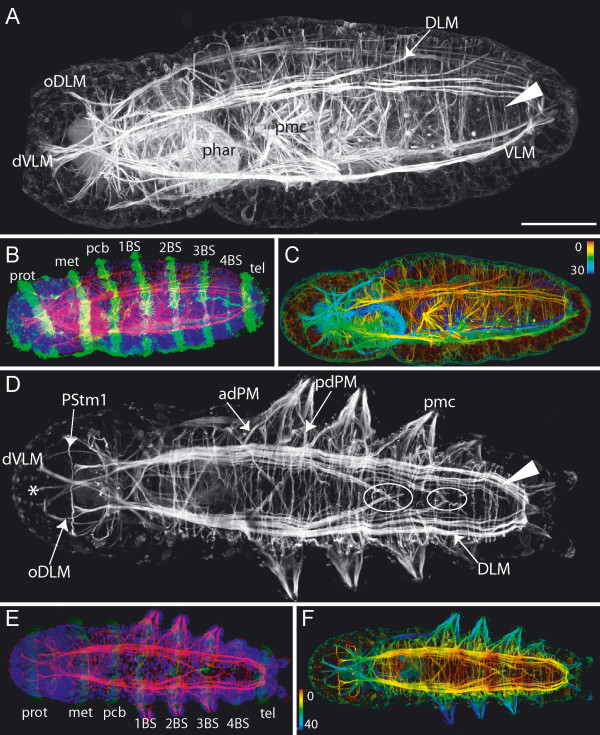
**Three to four chaetigerous ciliary bands: dorsal and lateral view**. Scale bars = 50 μm, anterior is to the left. **A-F**. Phallodin staining (**B, E **also include anti-acetylated tubulin staining in green and nuclear staining in blue). Prototroch (prot), metatroch (met), ciliary band of peristomium (pcb), ciliary band of chaetigers (BS) and telotroch (tel). **C, F**. Depth coded (in μm). **A-C**. Lateral view. In addition to the diagonal ventral longitudinal muscle (dVLM), the outer dorsal longitudinal muscle (oDLM) now extends into the prostomium. The pharynx (phar) is ventrally situated. The ventral longitudinal muscle (VLM) elongates straight posteriorly, whereas inner branches of the dorsal longitudinal muscle (DLM) run dorsomedially toward the midline. The arrowhead marks a circle of transverse muscle anterior to the pygidium. **D-F**. Dorsal view. Anteriorly, the outer dorsal longitudinal muscles (oDLM) and the ventral diagonal muscle (dVLM) connect within the prostomium, although the median projection of the dVLM (asterisk) remains weakly developed. Inner branches of the dorsal longitudinal muscle (DLM) intersect at the dorsal midline (circle). The anterior (adPM) and posterior dorsal parapodial muscles (pdPM) extend from the dorsal side into the parapodial muscle complex (pmc). The arrowhead marks the transverse muscle circle within the region anterior to the pygidium.

From here, the main events in myogenesis prior to hatching are the continued development of the prostomial muscles and the formation of the parapodial muscles in the first four chaetigers. The most prominent muscles of the prostomium are still the diagonal ventral longitudinal muscles (dVLM) (Fig. 7A–B and Fig. 8A). The middle branch of this muscle remains relatively thin, while the outer branches increase in thickness. They have already formed the connection with the straight ventral and the outer dorsal longitudinal muscles (Fig. 8D). However, the latter are only weakly developed at this stage, and the anterior muscle cap is not completely developed before hatching.

As all four embryonic chaetigers develop in an anterior-to-posterior progression, it is possible to examine different stages of parapodial development in one specimen. Initially, the anlage of the parapodia can be distinguished by the presence of autofluorescent chaetae and aciculae (Fig. 7A,B). The first parapodial muscles are the inner anterior and posterior muscles (iaPM, ipPM). Together with the outer and dorsal parapodial muscles (oaPM, opPM, adPM, pdPM) these muscles elongate into the distal tip of the parapodium, forming a conical array. Aciculae and chaetae are closely apposed within the proximal portion of the parapodium. Here, the chaetal and acicular muscles begin their development in direct contact with both types of bristles. During further parapodial development, the different bristle types are pulled apart as the acicular muscles shift toward the ventral midline.

## Discussion

This work is part of a series investigating annelid myogenesis with respect to its implications for the ground pattern of annelid musculature [6,7]. While a variety of patterns in adult polychaete musculature have been described elsewhere [30-34,37,44], the emphasis of the present analysis is on polychaete myogenesis, as polychaete and clitellate musculatures become more readily comparable when examined in a developmental context.

### Longitudinal and Prostomial Muscles

*Ophryotrocha diadema*'s adult musculature displays several features that have been described previously for many polychaete families, reviewed in [33]. The presence of one prominent pair of ventral longitudinal muscles plus a smaller longitudinal muscle situated just dorsally of the ventral nerve cord is common in investigated polychaete species. Conversely, the number and size of dorsal longitudinal muscles is more variable among taxa. In *O. diadema*, the dorsal longitudinal muscles comprise two dorsolateral bands separated by a considerable dorsomedian gap. The innermost fibers of each dorsal longitudinal muscle (DLM) repeatedly cross over each other at the dorsal midline along the anterioposterior axis, forming a regular pattern of intersecting dorsal longitudinal muscles distinct from the dorsal diagonal muscles (Fig. 1A, DLM, dm). To our knowledge, this muscle pattern has not yet been described in any other polychaete species, although it does appear in other members of the genus, such as *Ophryotrocha labronica *(J. Brubacher, unpublished observations).

These dorsal and ventral longitudinal muscles, together with a muscle ring surrounding the mouth opening, are the first muscles to differentiate during embryogenesis of *O. diadema*. Their formation starts within the peristomium, adjacent to the stomodeum. They gradually elongate towards the posterior by the addition of muscle fibers at their posterior end – a posterior growth zone. During this process, the longitudinal muscle strands do not display any sign of a repetitive pattern with respect to the body-segments.

Early events in the formation of longitudinal muscles have been also described in the polychaete *Capitella *sp. I [45]. Like *O. diadema*, *Capitella *sp. I lacks a free-living trochophore larva [40,46,47]. The onset of muscle formation in this species is characterized by the appearance of one pair of ventrolateral and four dorsal longitudinal muscles, which, as in *O. diadema*, elongate at their posterior ends. In *Capitella*, additional (secondary) longitudinal muscles are added after differentiation of these primary longitudinal muscles, forming an almost complete longitudinal muscle layer.

Although adult oligochaetes possess a homogeneous inner layer of longitudinal muscles, without distinct muscle bands [34,37] the formation of longitudinal muscles in oligochaetes parallels the patterns described above in important ways [6,7]. For example, myogenesis in *Enchytraeus coronatus *(Enchytraeidae) and *Limnodrilus *sp. (Tubificidae) begins with the formation of dorsal and ventral bilateral pairs of primary longitudinal muscles [6,7]. The ventral muscles arise adjacent to the developing ventral nerve cord – the same position occupied by the ventral longitudinal muscle bands in polychaetes [33]. As in *O. diadema *and *Capitella *sp. I, longitudinal muscles in these oligochaetes elongate from a posterior growth zone. During a second step in muscle differentiation, additional longitudinal muscle fiber strands are added dorsally and laterally (and ventrally in *Limnodrilus *sp.), completing the longitudinal muscle layer. The secondary longitudinal strands are always formed adjacent to an already-present primary or secondary longitudinal muscle and display the same mode of elongation towards posterior as described for the primary longitudinal muscles. By the end of embryogenesis, the initial primary muscles strands cannot be distinguished from the secondarily formed ones. This second step in development of the longitudinal muscle layer resembles the events during late myogenesis in *Capitella *sp. I, which possesses an oligochaete-like near-homogenous layer of longitudinal muscles [45]. This stage of secondary muscle formation is missing in *O. diadema*. Here, the initial ventral muscles retain their status as distinct muscle strands, and remain completely separated. The medially crossing strands of the dorsal longitudinal muscles (DLM, Fig. 1A) reflect a modification of the primary muscle growth pattern. However, we do not interpret this crossing as secondary muscle growth, as these strands form by branching of the primary dorsal muscle bands during their growth, rather than by formation of new, distinct muscle strands after the primary musculature has been laid down, as in oligochaetes.

While the number of primary dorsal longitudinal muscles varies among analyzed species (*Capitella *sp. I has four dorsal muscle strands, while *O. diadema*, *E. coronatus*, and *Limnodrilus sp*. each have only two) the presence of a pair of primary ventral longitudinal strands seems to be a common pattern among polychaetes and oligochaetes, irrespective of the adult musculature [34].

While the process of longitudinal muscle formation is similar in polychaetes and oligochaetes, leeches (Hirudinea), however, have their own characteristic pattern of embryonic muscle development. During myogenesis in the hirudinean *Erpobdella octoculata *(Erpobdellidae) no distinct primary longitudinal muscle bands are formed [6]. In this case, primary longitudinal muscles consist of thick bundles that branch at both ends. These primary muscles are scattered over the flanks of the embryo in an irregular manner, rather than in a stereotypical pattern of a set number of dorsal and ventral bands. Later, secondary longitudinal muscle strands emerge and grow in a gradual ventral-to-dorsal and anterior-to-posterior progression. The primary muscles are incorporated into the expanding lattice of secondary muscles to form the longitudinal muscle layer thereby. However, leeches deviate morphologically from other annelid groups in many ways, and are considered to represent a derived annelid taxon [35,48-51]. Therefore, the differences in their muscle formation relative to oligochaetes and polychaetes may also be a derived feature of this group [6].

Another feature of the ventral longitudinal muscles shared by polychaetes and oligochaetes is their major contribution to the musculature of the prostomium, which has been documented in a variety of annelid taxa. In the polychaetes *Nerilla antennata*, *Nerillidium *sp. and *Trochonerilla mobilis *(Nerillidae), the ventral longitudinal muscles extend into the prostomium, where they project into the median antennae [32]. For some polychaetes – i.e., *Magelona *cf. *mirabilis *(Magelonidae) and *Prionospio cirrifera *(Spionidae) – these ventral muscles are the only longitudinal strands that contribute to the prostomial musculature [30]. In *E. coronatus *and *Limnodrilus *sp. the ventral longitudinal muscles also elongate into the prostomium, where they each branch into two strands, and surround the anterior pole in a simple loop [6,7].

The dorsal longitudinal strands generally play a lesser part in the prostomial musculature, stretching straight anteriorly if they extend into the prostomium at all. During myogenesis in *E. coronatus *and *Limnodrilus *sp., the longitudinal muscles only grow into the prostomium after they are well-established within the trunk [6,7]. In *O. diadema*, the ventral muscles form three small projections that lie anterior to the mouth opening. They are the anlage of the diagonally running ventral prostomial muscles (dVLM), which expand towards the anterior pole during embryogenesis (Fig 6C), and persist after hatching (Fig 2C). The straight ventral prostomial muscles (sVLM) form later during embryogenesis. At first, these remain in a lateral position, but later develop anterior connections to the diagonally running ventral prostomial muscles (dVLMs) (Fig 7A,B).

The early state of prostomial muscle arrangement observed here for *O. diadema *is very similar to the one described for the adult muscle pattern in *Dorvillea kastjani *(Dorvilleidae, Polychaeta) [31]. In this species the three projections of the diagonal ventral longitudinal muscles (dVLM) (named by Filippova *et al*. pr/cr: prostomial cross and pr/r: prostomial rostral) are well developed but have no obvious anterior connection to the straight ventral longitudinal muscles (sVLM) (called pr/l: prostomium longitudinal by Filippova *et al*). In post-hatch *O. diadema*, however, the prostomial musculature continues to develop, such that the anterior connections between the dVLMs and sVLMs strengthen, and merge with the outer dorsal longitudinal muscle (oDLM) to form an anterior muscle cap. Development of the medial projection of the diagonal ventral longitudinal muscle (dVLM) is difficult to follow but it likely becomes the ventrally situated elongation of the dVLM depicted in Figure 2C (circle).

Our findings, combined with previous studies, suggest that a bilateral pair of ventral longitudinal muscles contributing to the prostomial musculature is a common feature in polychaetes and oligochaetes. The precise number of dorsal longitudinal strands included in this general muscle arrangement has yet to be determined; however two dorsal muscles seem to represent the most common pattern [34].

### Parapodial muscles

The majority of the body musculature, aside from the longitudinal muscles, is contained within the parapodial muscle complexes. The parapodial muscles in *O. diadema *are almost identical to those described for *Dorvillea kastjani *(Dorvilleidae) [31]. Interestingly, several investigations of polychaete parapodial muscles indicate that parapodial muscles of greatest complexity are found in species that do not use their appendages for locomotion [33,52]. In these species, the parapodia bear dorsally directed chaetae and likely function as defensive equipment. *O. diadema*, on the other hand, uses its uniramous, unornamented parapodia for locomotion in a walking-like manner, consistent with the relatively simple parapodial musculature described here. The four anteriormost parapodia are formed during embryogenesis in an anterior-to-posterior sequence, before the worm hatches from its egg case. After hatching, additional parapodia-bearing body segments are added through the activity of a proliferative zone just anterior to the pygidium, as in other annelids [53,54]. Although *O. diadema *lacks a trochophore larva, the timing of myogenic events in the trunk and parapodia during development resemble that of indirectly developing polychaetes with a free-living trochophore larva. These polychaetes, such as *Platynereis dumerilii *(Nereididae) and *Pomatoceros lamarckii *(Serpulidae), exhibit an early development of synchronously formed three bilateral pairs of parapodia, defining the metatrochophoral state [3,43]. Initially, both of these species have three parapodial segments. *P. lamarckii *undergoes metamorphosis to a sessile lifestyle, retaining the musculature of the larval parapods, whereas *P. dumerilii*, adapting the benthic lifestyle of the adult, grows by segment proliferation. Conversely, the temporal pattern in parapodial muscle differentiation is quite different in *Capitella *sp. I [45,46,55]. Chaetal, oblique and intrasegmental muscles are added during metamorphosis, after embryogenesis and the formation of the circular and longitudinal muscles of the body. This kind of change in the relative timing of developmental events in related taxa (heterochrony) is widely held to be an important mechanism underlying morphological change during evolution [43,56].

The earliest signs of parapodia in *O. diadema *are the chaetae, which lie just below the body-wall, roughly marking the middle of each body-segment. The first muscles of the parapodial complex to appear are the posterior parapodial muscles (pPM). These muscles occupy the same position as the transverse muscles (tm) anterior and posterior to the ciliary bands described for earlier stages Fig. 5E,H). Transverse muscles can easily be mistaken for true circular muscles at first glance. But since they lie below the longitudinal muscles, they do not possess the characteristic supralongitudinal position of true circular fibers, which always lie just below the epidermis (Fig. 6B). So it is possible that these transverse muscles are the anlage of the posterior parapodial muscles (pma). It might also be that the parapodial muscles cover the transverse muscles during their enhanced development. But this seems rather unlikely, because the close inspection of the adult parapodial muscle complex through 3D reconstruction did not reveal any other transverse muscles at this position, aside from inner and outer posterior parapodial muscles. The only other transverse muscles found on the ventral side are situated within the anterior halves of the body segment, without any connection to the posteriorly situated ciliary bands.

Chaetal and acicular muscles begin to appear only after the outer parapodial muscles started to form. Both the chaetal (chm) and acicular muscles (am) differentiate in direct contact with the chaetal types they support. It seems that the chaetae move into the distal portion of the growing parapodia and the aciculae migrate towards the midline, as their associated muscles lengthen (am and chm).

### Circular muscles

The segmentally arranged parapodial muscle anlagen (pma) that run in a transverse manner in close proximity to the ciliary bands do not represent true circular muscles, due to their position below of the longitudinal muscles. There are numerous transverse muscles present at the dorsal side of *O. diadema*. Some of these muscles belong to the parapodial muscle complex (adPM, pdPM) and lie below the dorsal longitudinal muscles. However, there are also transverse muscles in a supralongitudinal position, which could be clearly identified in histological sections (Fig. 1E). These fibers are only weakly developed, and we could not determine whether they form complete circles surrounding the whole body of *O. diadema*. Complete circular muscles must be rare, if present at all, as only a few ventral transverse muscles could be found, whereas there are plentiful transverse muscles present dorsally and laterally. This discrepancy implies that many incomplete circular muscles exist, spanning the dorsal side but terminating at the laterally in chaetigerous segments. Such a patterns corresponds with the findings in *Dorvillea kastjani*, where true circular fibers were only present in the intersegmental furrows [31]. Interestingly, several complete rings of transverse muscles exist in the zone of segment proliferation, just anterior to the pygidium, in juvenile *O. diadema *(Fig. 1A and 8D), some of which are true circular muscles (*i.e.*, external to the longitudinal muscles). This pattern resembles that described for regenerating fragments in *Dorvillea bermudensis *[34]. In *D. bermudensis*, closed circles of transverse fibers are established initially in the regenerating region, but are later disrupted by formation of parapodia. This pattern observed in regenerating specimens may therefore represent a juvenile character.

## Conclusion

Recent studies of polychaetes have revealed a great variety of adult muscle patterns, unlike the closed, homogeneous muscle layers present in oligochaete taxa. These obviously different arrangements have considerably hampered attempts to directly correlate the musculatures of these groups with each other, in the ongoing discussion concerning the ground pattern of annelids. However, developmental data from various annelid taxa has revealed common characters evident during myogenesis in these species. Here, we have shown that *Ophryotrocha diadema *shares with the oligochaetes *Enchytraeus coronatus *and *Limnodrilus *sp. distinct ventral longitudinal muscle bands that are almost identically established during ontogenesis. We consider it very likely that these muscular structures are homologous, and that the primary formation of a bilateral pair of ventral longitudinal muscles is an ancestral character of annelids.

Unfortunately, due to the lack of a reliable annelid phylogeny, we can only speculate about the direction of evolutionary change in annelid musculature. Taking the presence of a bilateral pair of ventral longitudinal muscles in early development as an ancestral character, it follows that the oligochaete-like musculature and embryonic formation of secondary longitudinal muscles is either an evolutionary novelty (and therefore derived) or a character that has been lost in polychaetes. In the latter case, the loss of secondary muscle formation in polychaetes would be the derived condition.

## Methods

### Worm culture

Cultures of *Ophryotrocha diadema *(a gift from Bertil Åkesson, Göteborg University) were maintained in glass dishes at room temperature, in artificial sea water (Crystal Sea Marinemix, Marine Enterprises Intl., Baltimore, MD) with a specific gravity of 1.021 g·ml^-1^. Each week, half the seawater in the dishes was refreshed, and worms were fed spinach finely chopped with a Waring blender.

### Fixation

Unless otherwise noted, all chemicals used in fixation were from Sigma, Oakville, Canada. *O. diadema *brood their embryos in transparent, gelatinous tubes. Worm embryos and larvae were removed from brood tubes using 0.1 mm minuten pins mounted on applicator sticks. We fixed them in seawater + 4% (w/v) paraformaldehyde (Electron Microscopy Sciences, Hatfield, PA), for either 3 h at room temperature, or overnight at 4°C. Larvae mature enough to show evidence of muscle activity were anaesthetized for 10 min in a 1:1 mixture of seawater and 370 mM MgCl_2 _prior to fixation. After fixation, we washed worms several times in phosphate-buffered saline, pH 7.4 (PBS; 137 mM NaCl, 2.7 mM KCl, 10 mM Na_2_HPO_4_, 1.4 mM KH_2_PO_4_) plus 0.1% (v/v) Tween-20 (PBT). Prior to use, worms were stored at 4°C in PBT + 0.05% (w/v) sodium azide.

### 3D modeling of musculature from serially sectioned, epoxy-embedded material

For histological analysis, worms were anaesthetized in a mixture of equal parts sea water and 370 mM MgCl_2_, then fixed for 2 days at 4°C in 3% (w/v) glutaraldehyde + 0.5% (w/v) paraformaldehyde, in a buffer containing 100 mM PIPES, 2 mM EGTA, 1 mM MgSO_4_, and 200 mM sucrose, pH 6.9. After primary fixation, worms were washed in 0.1 M sodium cacodylate, pH 7.2, then further fixed for 90 min in 1% (w/v) OsO_4 _in washing buffer, on ice. Specimens were dehydrated through graded ethanol into acetone, and embedded in a 4:5:12 mixture of Araldite 502, EMbed 812 and dodecenyl succinic anhydride, catalyzed with 1.5% (v/v) DMP-30. (Fixatives and resin components from Electron Microscopy Sciences, buffering agents and salts from Sigma).

Serial 1 μm transverse sections were cut from tissue blocks on a Porter-Blum MT-2B ultramicrotome, and stained with a mixture of methylene blue and azure B, followed by basic fuchsine [57]. Grayscale images of sections illuminated with red, green and blue light were captured and merged to generate color images, using an AxioImager Z.1 microscope equipped with an AxioCam MRm camera and AxioVision 4.6 software (Zeiss). To align images, trace features of interest, and construct 3D models from serial sections, we used IMOD software, v. 3.5.3 [58].

### Immunostaining

For immunostaining, a protocol previously described for *Enchytraeus japanoensis *was adapted [59]. Incubation times within the described buffers were reduced to two to three hours each, depending on embryonic stage of the specimens. A monoclonal anti-acetylated tubulin antibody (Sigma, T-6793) was used at 1:500 dilution in 1 × PBS (140 mM NaCl, 6.5 mM KCl, 2.5 mM Na_2_HPO_4_, 1.5 mM KH_2_PO_4_, pH 7.5) at 4°C for 12 to 48 hours. After several washes in PBT (PBS + 0,1% (v/v) Tween-20), the secondary antibody (anti-mouse Cy2 conjugated; Dianova, Hamburg, Germany) was applied diluted 1:150 in PBS for 12 to 48 hours at 4°C. Unbound antibody was removed by several washes with PBT.

### Phalloidin staining

After immunostaining, the embryos were transferred into a freshly made solution of TRITC-conjugated phalloidin (Sigma, P1951) prepared by evaporating 5 μl of a stock solution (3.3 μM in ethanol) and reconstituting in 250 μl of PBS. Embryos were stained in this solution for 1–3 h, in the dark. The phalloidin solution was then removed and the embryos were washed several times with PBT.

### Nuclear staining

We used Draq5™ (Alexis Biochemicals, Germany) to label nuclei. After immuno – and phalloidin staining of *O. diadema *embryos, this dye solution was applied at a 1:500 dilution in PBS. After incubation for 30 minutes in the dark, the dye was removed, the embryos were washed several times in PBT, and mounted in Fluoromount-G (Southern Biotech, USA) before microscopic inspection.

### cLSM and 3D-Reconstruction

All fluorescence pictures were captured with a Pascal 5 Laser Scanning Microscope (Zeiss, Germany) using a He-Ne laser for TRITC and Draq5™ (543 nm and 633 nm, respectively) and an argon laser (488 nm) for Cy2 documentation. When multi-colored picture stacks were taken, the same plane thickness and distance were used for all staining conjugates to minimize optical shift. Digital pictures were edited with Adobe PhotoShop (San Jose, CA, USA) to adjust brightness and contrast. The same software was used to combine projections to generate complete montages of large embryos.

3D-reconstructions were carried out using amira™ 3.0 (Mercury Computer Systems, USA) using the label-field tool to individually label single muscles in each cLSM produced picture stack. 3D-Figures were obtained with the surface-generating algorithm of the software.

## Competing interests

The author(s) declare that they have no competing interests.

## Authors' contributions

AB conceived the study and wrote the first draft of the manuscript; conducted the fluorescent stainings, the cLSM analysis and the 3D reconstruction of the latter. JLB provided the animals, did the histological sections and the subsequent 3D reconstruction. AB and JLB interpreted results and wrote the manuscript. AP contributed substantially to the interpretation of data and to the writing of the manuscript. All authors read and approved the final manuscript.
